# Optimizing Guide Extension Catheter Advancement in Percutaneous Coronary Intervention: A Stepwise Approach Enhanced by Repeated Proximal to Distal Balloon Anchoring

**DOI:** 10.7759/cureus.87676

**Published:** 2025-07-10

**Authors:** Hasan Kazma, Hussam Haidar, Vincent J Berry, Malek Mohammed, Abbas Rachid

**Affiliations:** 1 Department of Cardiology, Bahman University Hospital, Beirut, LBN; 2 Department of Medicine, Faculty of Medical Sciences, Lebanese University, Beirut, LBN; 3 Department of Interventional Cardiology, Bahman University Hospital, Beirut, LBN; 4 Invasive Cardiac Laboratory, Bahman University Hospital, Beirut, LBN; 5 Department of Internal Medicine, Lebanese University, Beirut, LBN

**Keywords:** buddy coronary wire, calcified coronary artery, calcified coronary lesion, cutting balloon, drug-eluting stent, extra-backup guiding catheter, guiding extension catheter, primary percutaneous coronary intervention (pci), small-balloon anchoring technique

## Abstract

The advancement of drug-eluting stents (DES) to distal, severely calcified, and tortuous coronary artery lesions presents a significant challenge in interventional cardiology. Techniques such as extra-backup catheters, buddy wires, and guiding extension catheters are commonly employed to overcome these difficulties. When these conventional methods fail, the stepwise approach enhanced by repeated proximal to distal balloon anchoring helps advance the guiding extension catheter deep into the coronary artery, allowing the delivery of DES to the distal calcified lesions even in tortuous arteries, and serves as an effective bailout strategy. The stepwise approach enhanced by repeated proximal to distal balloon anchoring is used to overcome difficulties in DES delivery. It allows deep advancement of the guiding extension catheter into the coronary artery over the shaft of the anchored (inflated) balloon, facilitating DES delivery.

This case report details the percutaneous coronary intervention (PCI) of the right coronary artery (RCA) in a 69-year-old male patient with multiple severe diffuse calcified stenosis. Despite initial attempts with a Judkins right curve (JR4) 6 French guiding catheter (Medtronic, Inc., Minneapolis, MN) and a buddy wire technique, DES delivery was unsuccessful, given that the guiding catheter support was unstable when the DES was advanced into the artery. Thus, to facilitate DES delivery, a guiding extension catheter was used, but it failed to advance into the RCA. However, using the stepwise approach with repeated proximal to distal balloon anchoring allowed deep advancement of the guiding extension catheter into the RCA over the anchored (inflated) balloon shaft. This anchored balloon technique was done three times starting at the proximal part of the RCA and advancing the guiding extension catheter to proximal, mid, and finally distal part of the vessel, ultimately allowing the successful placement of three DES from the distal to the proximal part of the vessel, demonstrating the technique's efficacy in overcoming complex anatomical challenges. At the end of the procedure, all lesions were successfully stented with no residual stenosis and a thrombolysis in myocardial infarction (TIMI) III flow in the RCA, and the patient was stable hemodynamically with no chest pain.

## Introduction

Delivering a drug-eluting stent (DES) to a distal calcified coronary artery lesion is a frequent challenge in the cardiac catheterization laboratory [1]. Calcified lesions pose significant obstacles due to their rigidity, which hinders the passage of stents and other interventional devices. Traditional techniques to overcome this issue include extra-backup guiding catheters, buddy wires, and guiding extension catheters, which provide additional support and facilitate device advancement [1,2]. In cases where these methods prove inadequate, other approaches may be required: scoring balloons, cutting balloons, and super high-pressure balloons are used to prepare the heavily calcified lesion. Other techniques, such as ablative atherectomy, laser atherectomy, and intravascular lithotripsy, may be used to prepare the lesion. However, the use of these techniques is also limited by the ability to deliver them to the lesion. In fact, in case of severe calcification of the coronary artery before the lesion, the advancement of such devices is difficult and sometimes not feasible [2]. Intracoronary imaging technique, if available and technically feasible, such as optical coherent tomography (OCT) or intravascular ultrasound, may help in planning the percutaneous coronary intervention (PCI) strategy, including the use of atherectomy devices [1,2]. In cases where the techniques such as extra-backup guiding catheters, buddy wires, and guiding extension catheters are ineffective in delivering DES or other devices, a more innovative approach may be required. The next-generation guide extension catheters, especially the 4-5 French size (4-5 F) tapered guiding extension catheters used for PCI, can help advance devices to the lesion better than the old-generation guiding extension catheters [3-5]. A stepwise approach of advancing the guide extension catheter through the coronary artery is enhanced by repeated balloon anchoring that involves inflating a balloon at or beyond the lesion site to anchor it and then advancing the guide extension catheter over the shaft of this balloon, thereby allowing advancement of the guide extension catheter through the coronary artery and thus facilitating deployment of the stent or other devices to the lesion [6,7]. This technique leverages the mechanical advantage provided by the balloon's anchoring force, facilitating the progression of the guiding extension catheter through the calcified segment. We report a case demonstrating the efficacy of this technique in a patient with a severely calcified and tortuous right coronary artery (RCA), where other conventional techniques initially failed [6,7].

## Case presentation

**Figure 1:**
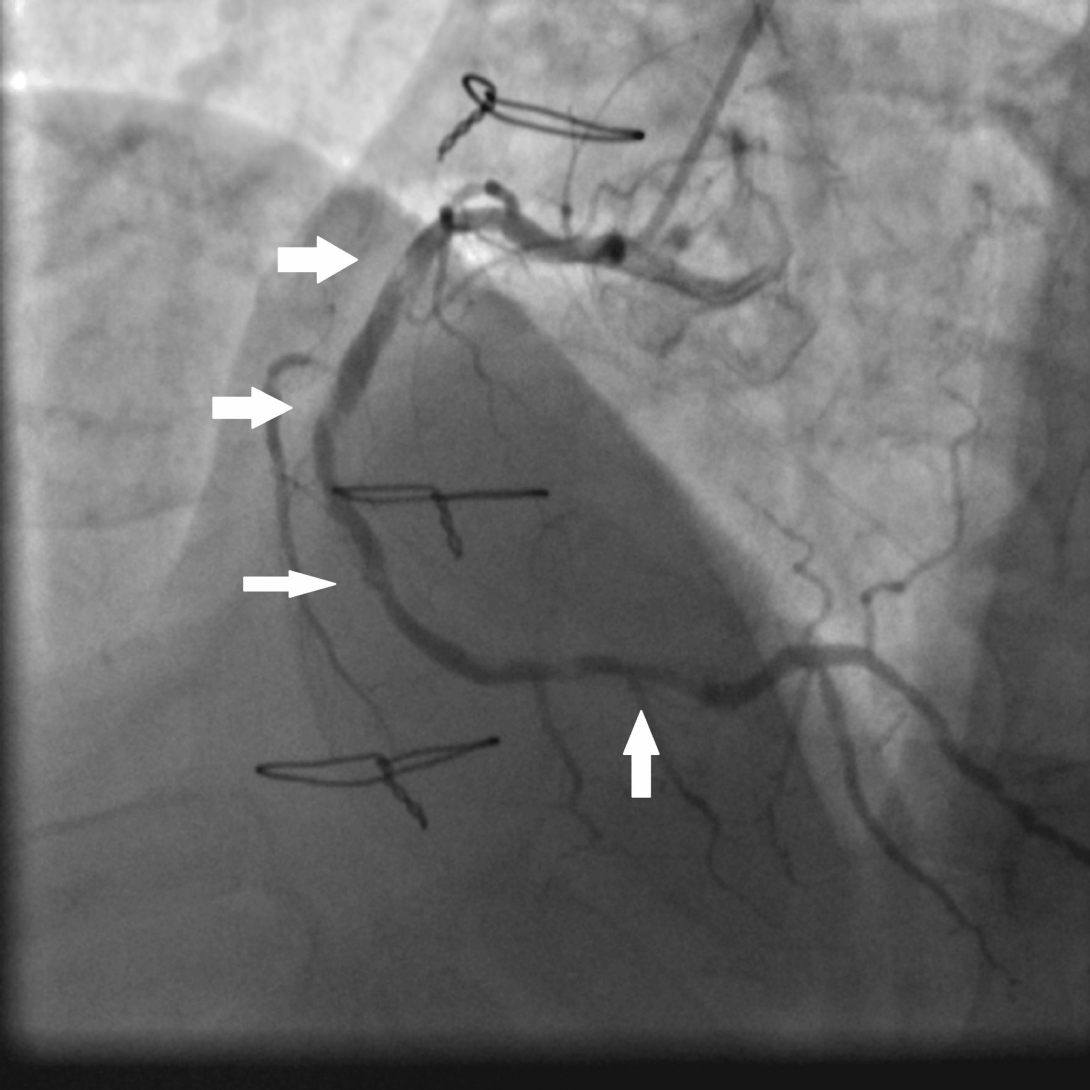
Coronary angiography showing multiple calcified tight stenosis of the proximal, mid, and distal RCA (white arrows). RCA: right coronary artery

A 69-year-old male patient presented to our hospital with angina during minimal physical activity. His medical history includes smoking, hypertension, dyslipidemia, and coronary artery disease. Ten years prior to presentation, he underwent a coronary artery bypass grafting procedure with a left internal mammary artery graft to the left anterior descending coronary (LAD) artery. He was on the following medications: aspirin 100 mg daily, valsartan 160 mg daily, bisoprolol 2.5 mg daily, and atorvastatin 40 mg daily. An echocardiogram showed a normal left ventricular ejection fraction at 55%, with no wall motion abnormalities or valvular disease. Given his symptoms, a coronary angiogram was performed, revealing a normal left main coronary artery, chronic total occlusion of the LAD artery, no significant stenosis in the circumflex artery, patent left internal mammary artery graft to the LAD artery, and severe, diffuse calcification with multiple severe stenosis in the right coronary artery (RCA). These were three consecutive heavily calcified lesions in the proximal, mid, and distal RCA, classified as class C according to the American College of Cardiology/American Heart Association (ACC/AHA) classification (Figure 1).

**Figure 2:**
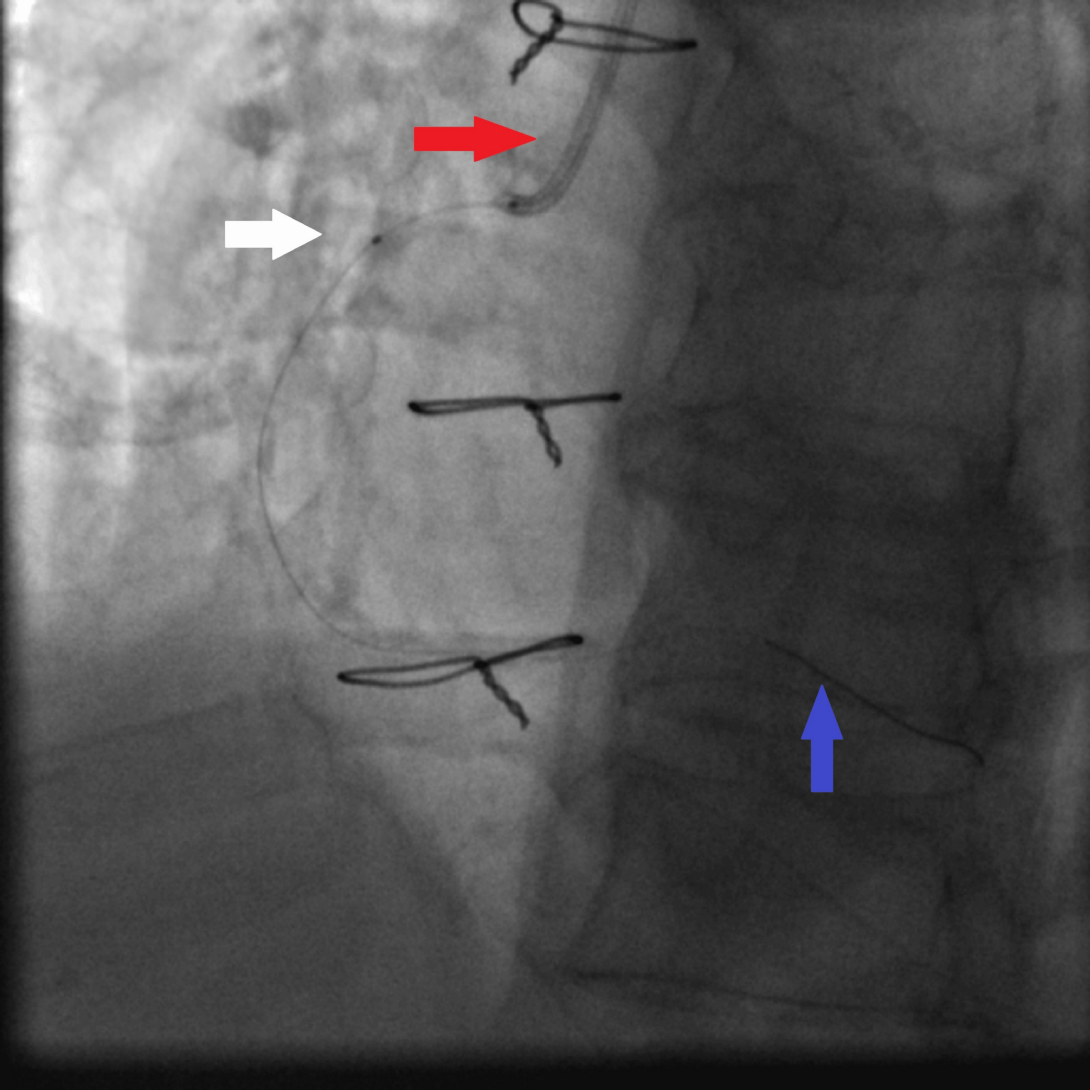
Fluoroscopy showing the JR4 6 French guiding catheter intubating the ostium of the RCA (red arrow) and the Asahi Sion coronary wire with its tip secured in the distal RCA (blue arrow); a small balloon (1.5×15 mm semi-compliant Solaris balloon) is seen advanced to the proximal RCA lesion (white arrow). RCA: right coronary artery, JR4: Judkins right 4

Based on these findings and the clinical symptoms, PCI of the RCA was indicated and performed. The patient received a 300 mg loading dose of clopidogrel, followed by a 75 mg daily dose. The RCA was cannulated using a Judkins right 4 (JR4) 6 French guiding catheter (Medtronic, Inc., Minneapolis, MN). The RCA lesions were crossed using an Asahi Sion coronary wire, and its tip was securely positioned in the distal RCA (Figure 2).

**Figure 3:**
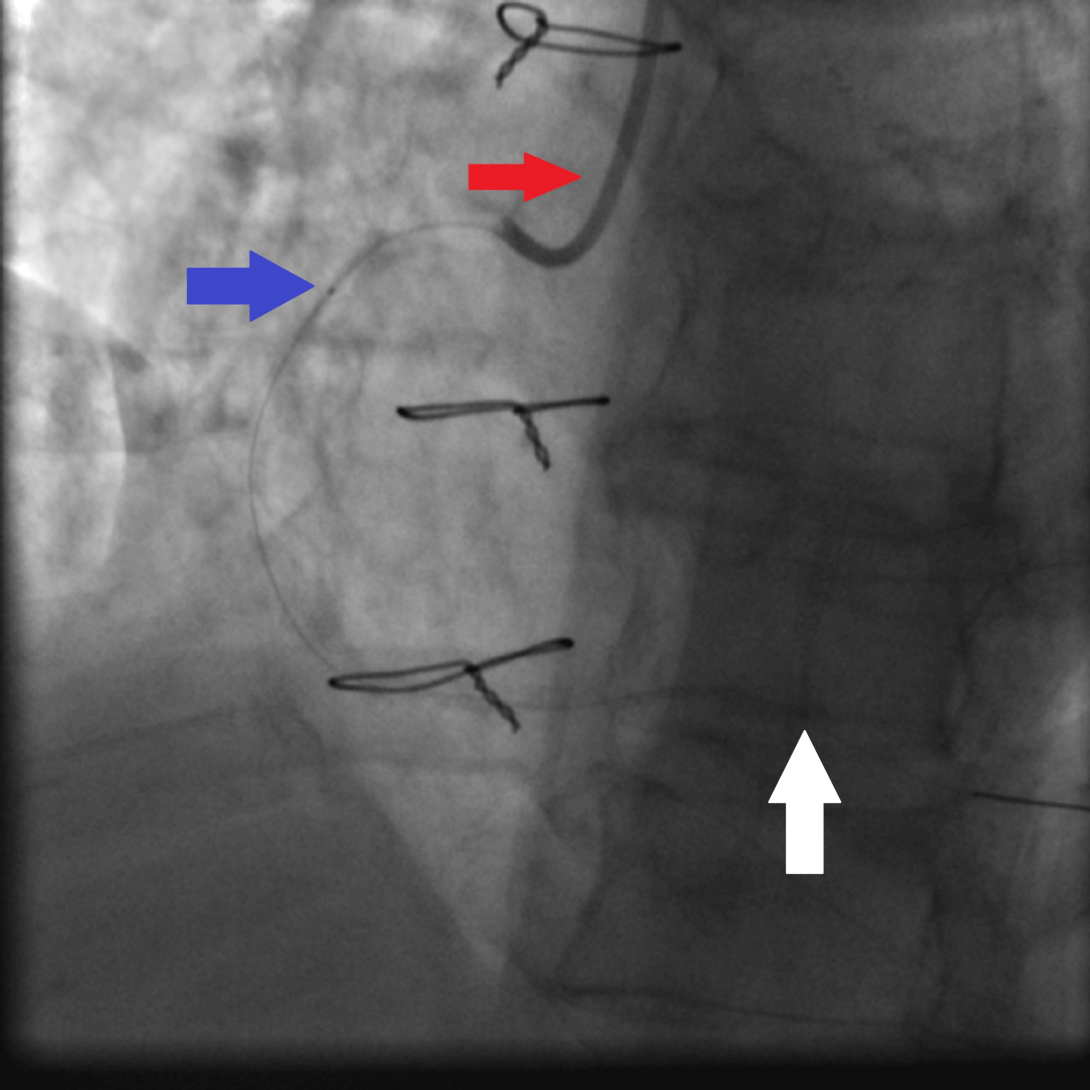
JR4 6 French guiding catheter (red arrow), small balloon (1.5×15 mm semi-compliant balloon) (blue arrow) inflated at high pressure (16 atmospheres), and distal Asahi Sion coronary guidewire secured in the distal RCA (white arrow). RCA: right coronary artery, JR4: Judkins right 4

**Figure 4:**
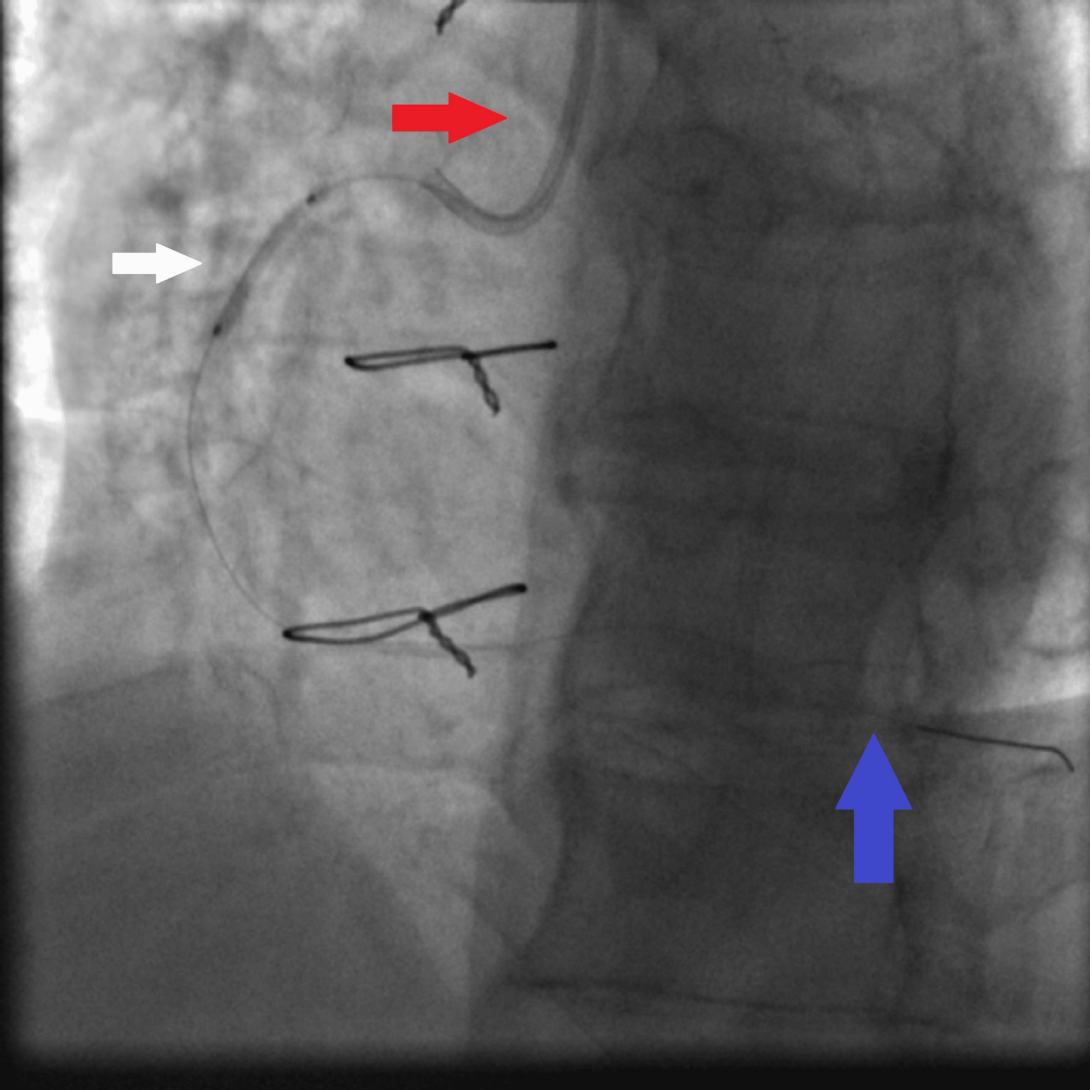
Fluoroscopy showing dilatation of the proximal RCA lesion using a 2.25×15 mm non-compliant Solaris balloon: JR4 6 French guiding catheter (red arrow), the 2.25×15 mm non-compliant Solaris balloon inflated at high pressure (16 atmospheres) (white arrow), and the tip of the Asahi Sion coronary wire secured in the distal RCA (blue arrow). RCA: right coronary artery, JR4: Judkins right 4

**Figure 5:**
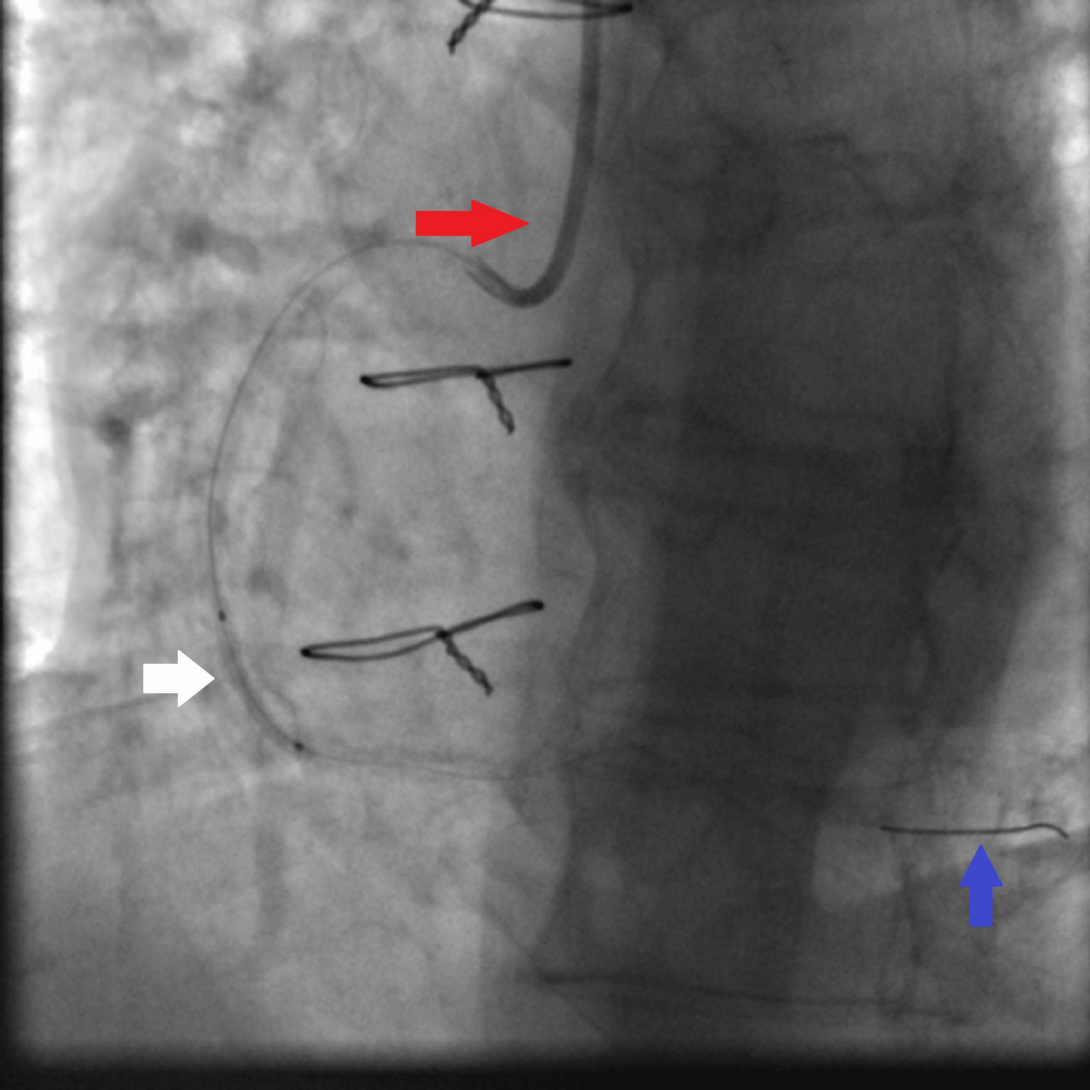
Fluoroscopy showing dilatation of the mid RCA lesion using the 2.25×15 mm non-compliant Solaris balloon: JR4 6 French guiding catheter (red arrow), the 2.25×15 mm non-compliant Solaris balloon inflated at high pressure (16 atmospheres) (white arrow), and the tip of the Asahi Sion coronary wire secured in the distal RCA (blue arrow). RCA: right coronary artery, JR4: Judkins right 4

**Figure 6:**
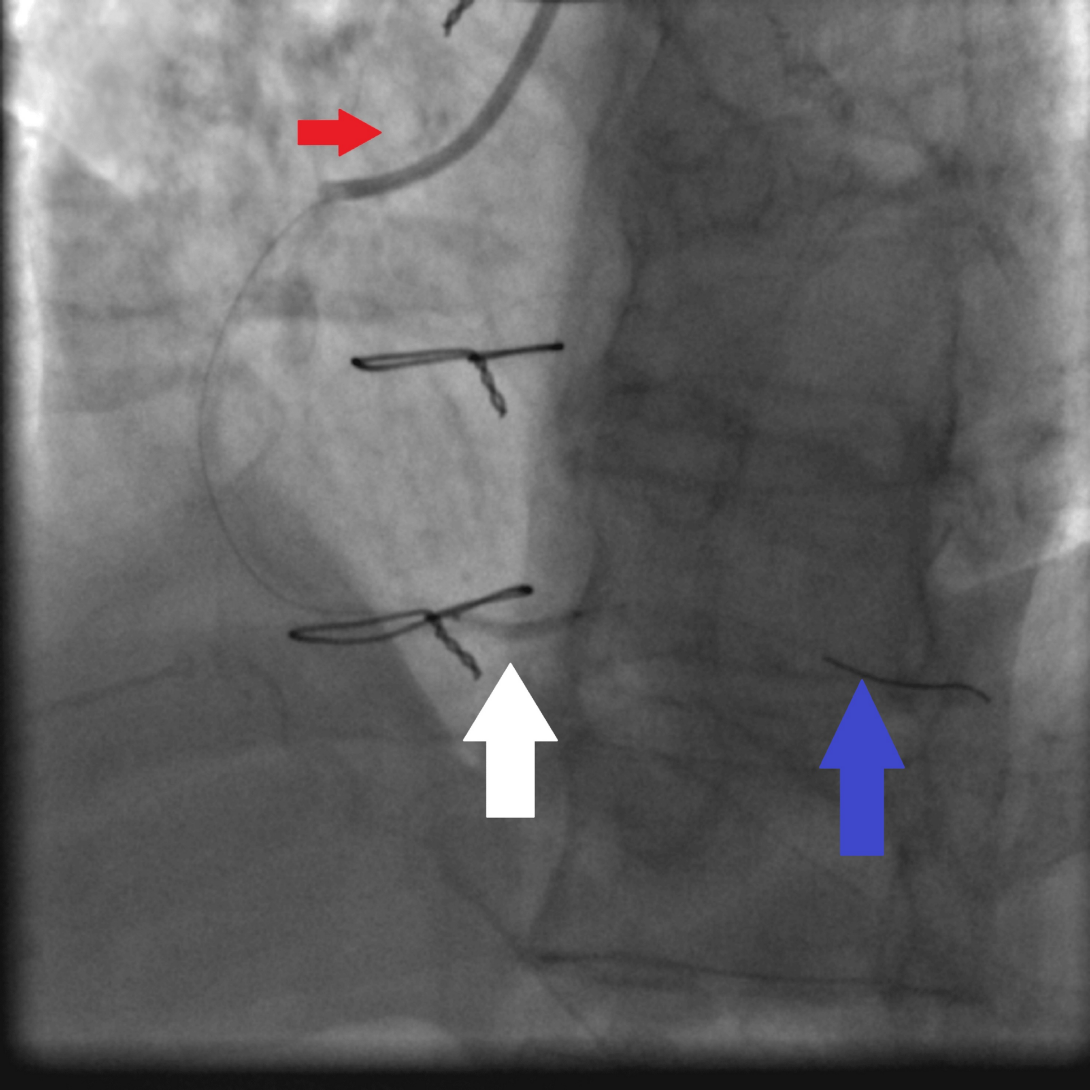
Fluoroscopy showing dilatation of the distal RCA lesion using a 2.25×15 mm non-compliant Solaris balloon: JR4 6 French guiding catheter (red arrow), the 2.25×15 mm non-compliant Solaris balloon inflated at high pressure (16 atmospheres) (white arrow), and the tip of the Asahi Sion coronary wire secured in the distal RCA (blue arrow). RCA: right coronary artery, JR4: Judkins right 4

Multiple balloon dilatations were performed at all lesion sites; the proximal lesion could only be crossed and dilated with a 1.5×15 mm semi-compliant Solaris balloon, and then, subsequently, all lesions were dilated using a 2.25×15 mm non-compliant (NC) Solaris balloon at high pressure (16 atmospheres). The operator did a step-up wise dilatation of all RCA lesions using first a small-sized balloon and then using a larger NC balloon because at first, only the small-sized balloon could cross the lesions. After the dilatation with the small-sized balloon, further dilatation with a bigger NC balloon was possible (Figures 3-6).

**Figure 7:**
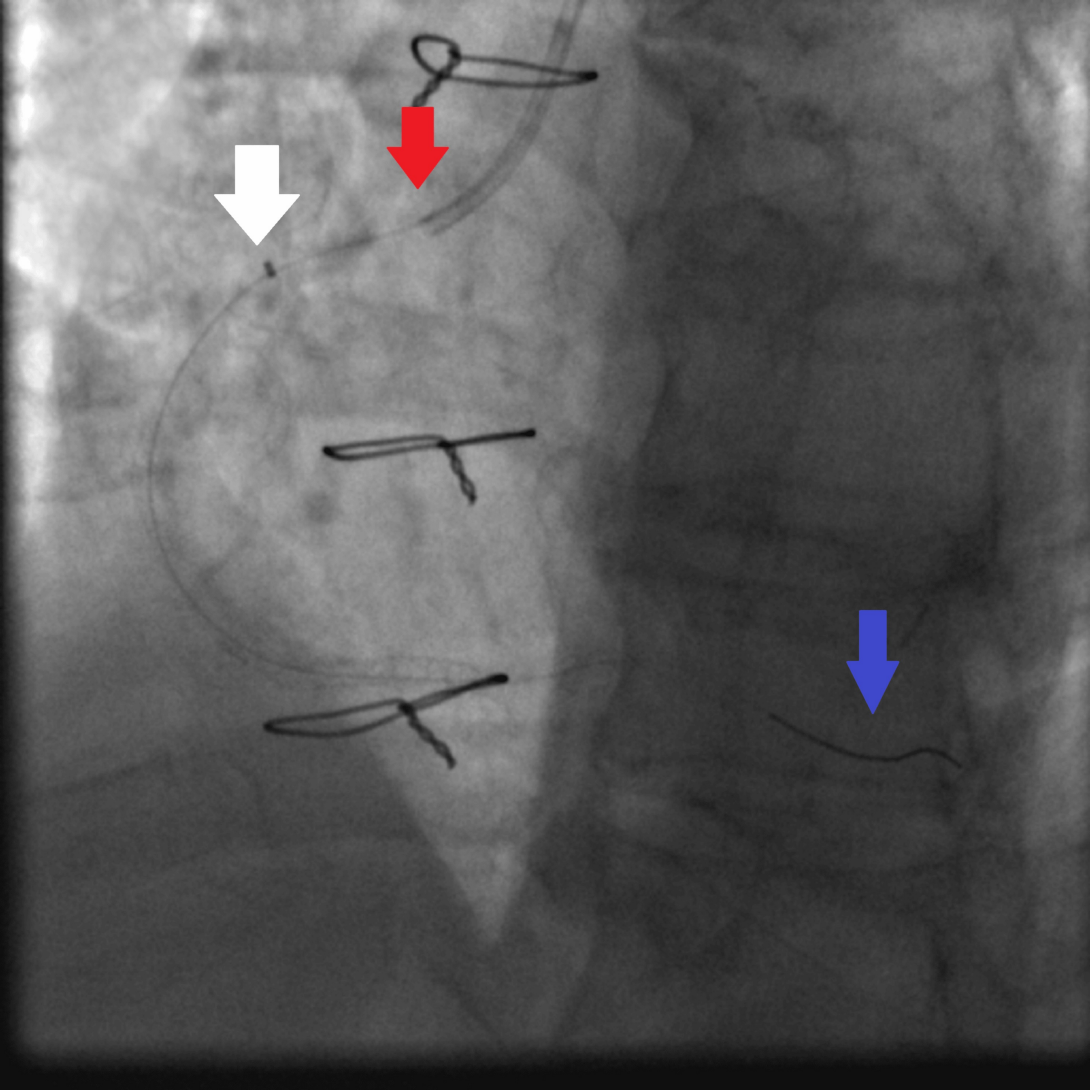
Fluoroscopy showing guiding extension catheter at the proximal RCA level: JR4 6 French guiding catheter (red arrow), proximal part of the guiding extension catheter at the proximal RCA site (white arrow), and the tip of the distal Asahi Sion coronary wire secured in the distal RCA (blue arrow). RCA: right coronary artery, JR4: Judkins right 4

Despite these efforts, the initial attempt to deploy a DES to the distal RCA lesion was unsuccessful, mainly due to the fact that the guiding catheter support was poor. So, a buddy wire (Balance MiddleWeight (BMW) coronary wire (Abbott, Abbott Park, IL)) was used, but the DES could not be advanced to the distal RCA lesion. The patient started to complain of chest pain. He remained hemodynamically stable with a blood pressure of 130/85 mmHg, and no electrocardiographic (ECG) changes were noted. A guiding extension catheter (Telescope Medtronic 6 French (Medtronic, Dublin, Ireland)) was utilized to extend the JR4 guiding catheter deeper into the RCA; due to the patient's chest pain, the operator opted not to switch to an extra-support guiding catheter due to time constraints and patient discomfort, instead proceeding with the guide extension catheter (Figure 7). Also, by changing the guiding catheter, the operator would have to lose the Asahi Sion wire already inside the RCA.

**Figure 8:**
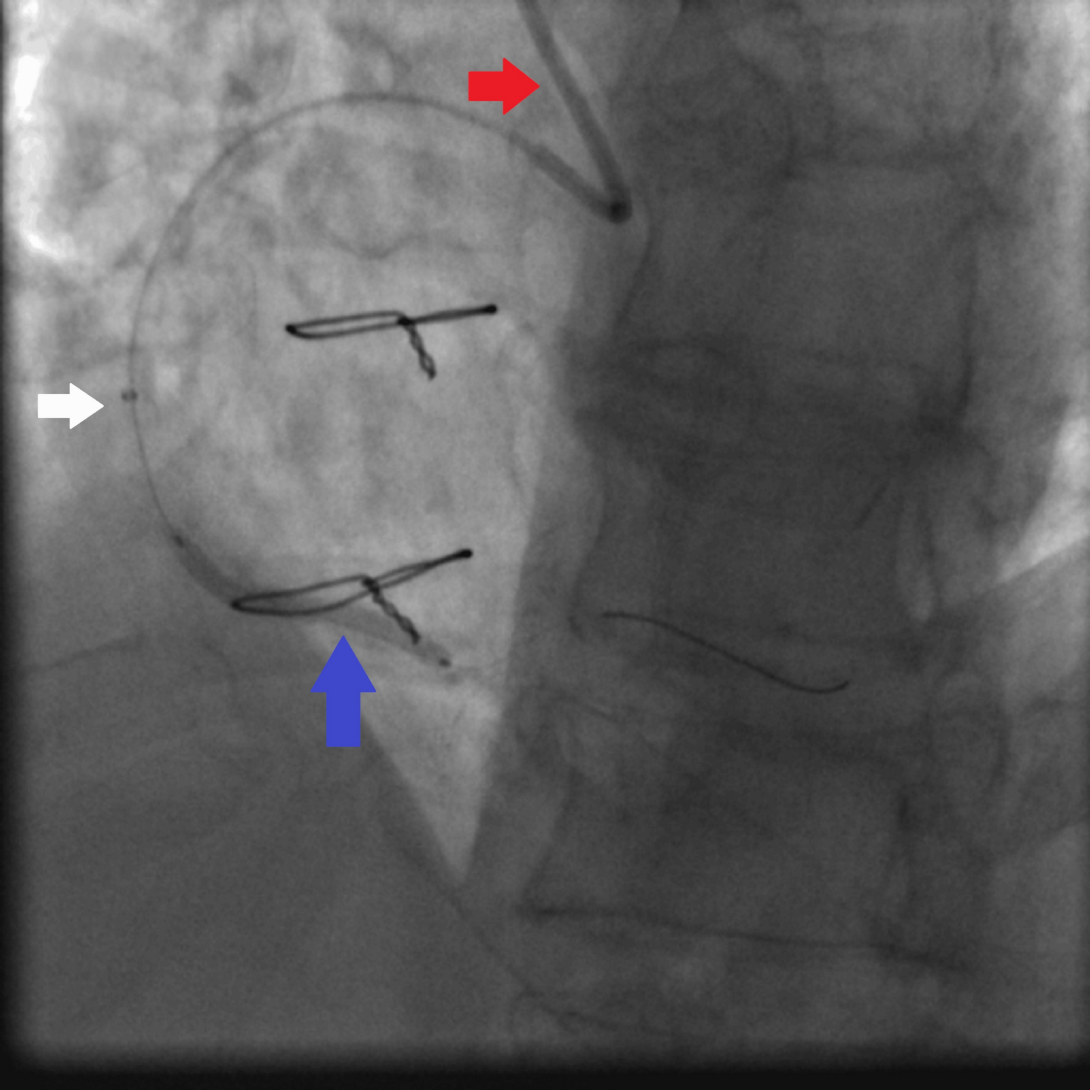
Fluoroscopy showing distal balloon anchoring in the RCA, allowing advancement of the guiding extension catheter over the balloon shaft to the mid RCA: JR4 6 French guiding catheter (red arrow), guiding extension catheter being advanced with its tip in the mid RCA (white arrow), and anchoring balloon inflated to facilitate advancement of the guiding extension catheter (blue arrow). RCA: right coronary artery, JR4: Judkins right 4

**Figure 9:**
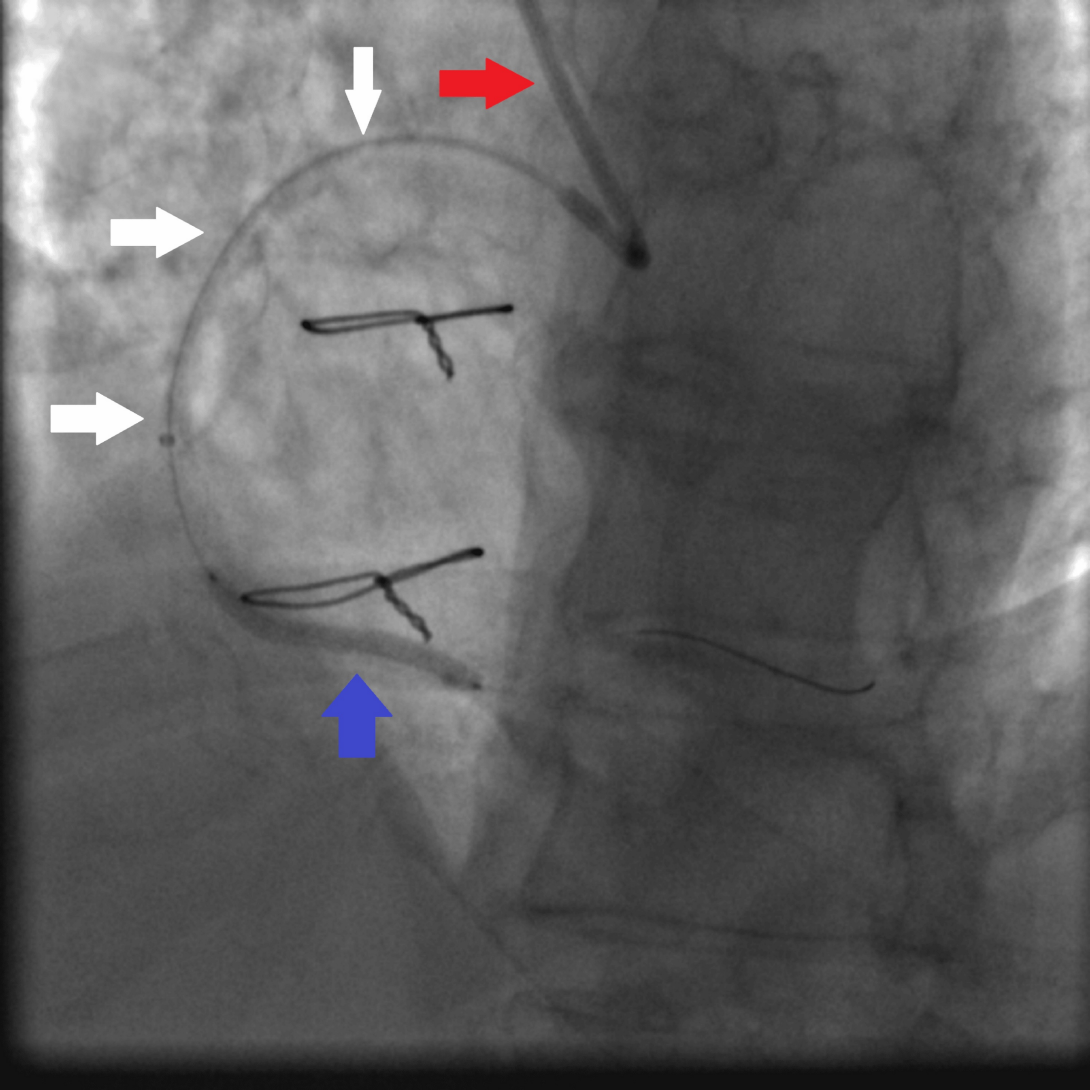
First distal DES (2.5×30 mm Orsiro DES) deployment facilitated by guiding extension catheter being advanced to the mid RCA: JR4 guiding catheter (red arrow), guiding extension catheter with its tip reaching the mid RCA (white arrows), and the first distal DES being deployed to the distal RCA lesion at high pressure (14 atmospheres) (blue arrow). DES: drug-eluting stent, RCA: right coronary artery, JR4: Judkins right 4

**Figure 10:**
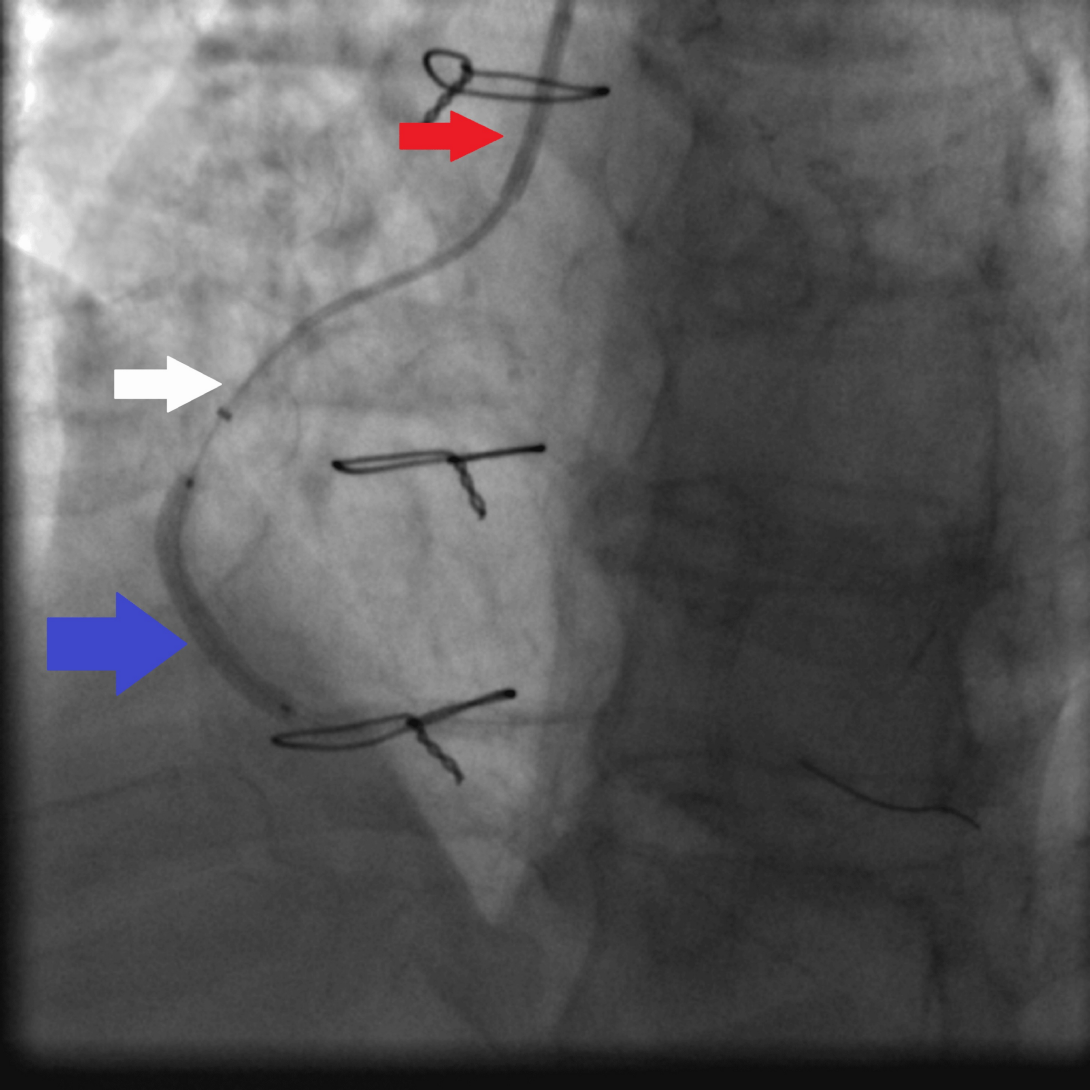
Second DES (2.5×30 mm Orsiro DES) being deployed to the mid RCA; this deployment is facilitated by the guiding extension catheter advanced deeply in the proximal RCA: JR4 6 French guiding catheter (red arrow), guiding extension catheter deeply intubating the proximal RCA (white arrow), and the second DES being deployed to the mid RCA lesion at high pressure (18 atmospheres) (blue arrow). DES: drug-eluting stent, RCA: right coronary artery, JR4: Judkins right 4

**Figure 11:**
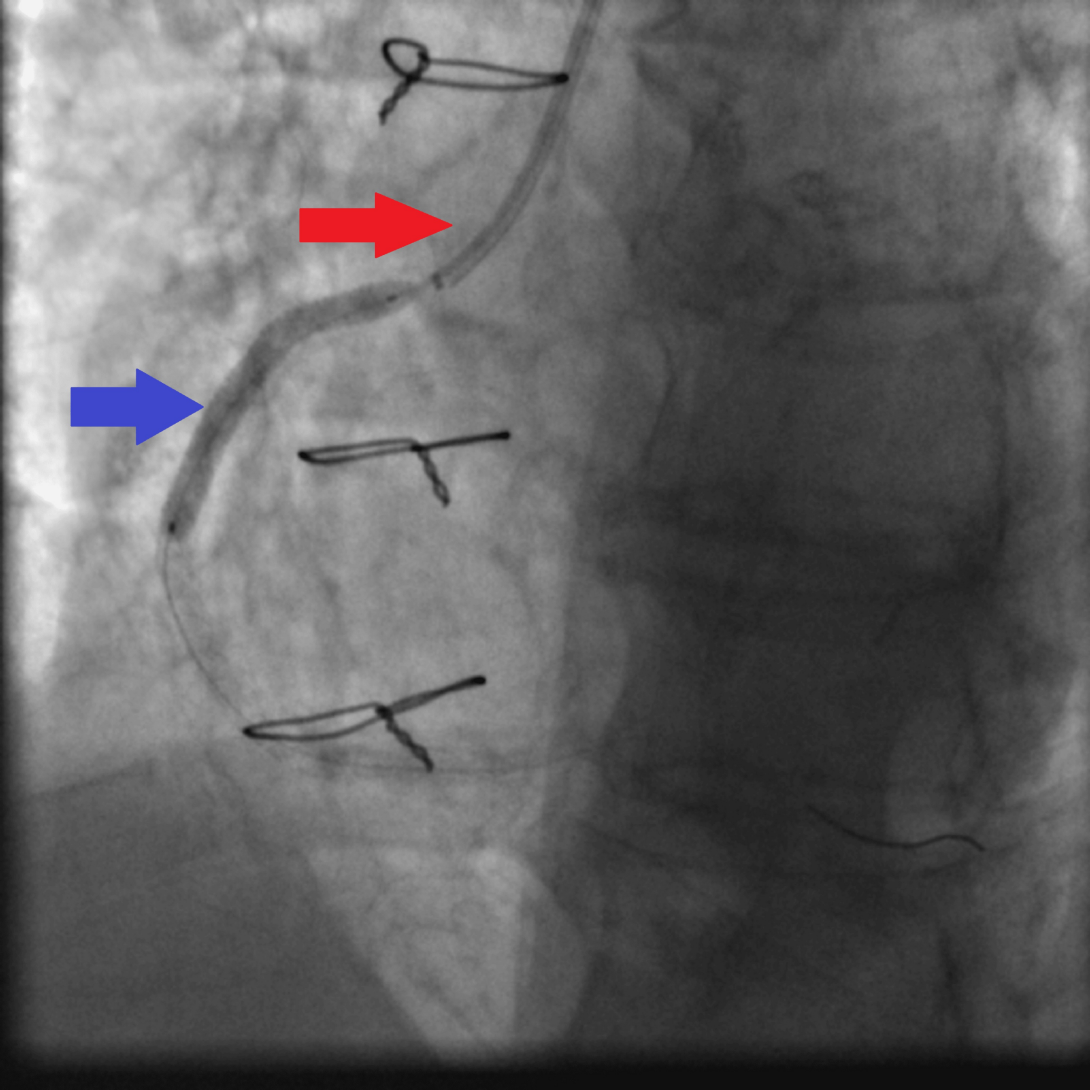
Third DES (3.5×34 mm Resolute Onyx DES) being deployed to the proximal RCA lesion; the guiding extension catheter was withdrawn into the guiding catheter: JR4 6 French guiding catheter (red arrow) and the third DES being deployed to the proximal RCA lesion at high pressure (16 atmospheres) (blue arrow). RCA: right coronary artery, JR4: Judkins right 4

**Figure 12:**
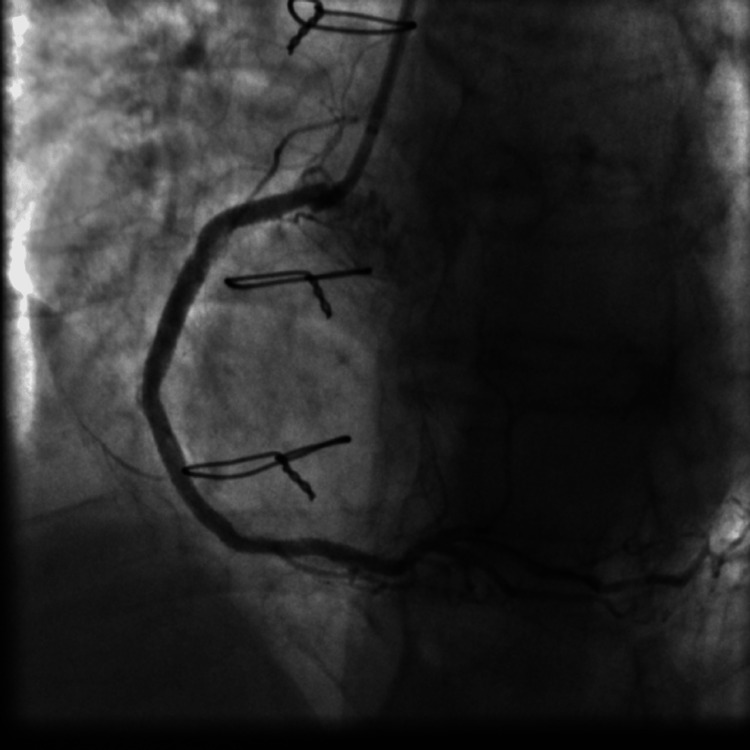
Final results after three DES placement showing good angiographic results with no thrombosis and no dissection in the vicinity of the three DES deployed. DES: drug-eluting stent

This technique also failed to deliver the DES, and the patient continued to experience angina. To address this, the balloon anchoring technique was employed. A 2.5×30 mm non-compliant Sprinter Legend balloon (Medtronic, Inc., Minneapolis, MN) was used initially to dilate the proximal, mid, and distal RCA lesions at 14 atmospheres, and as it was inflated in the vessel (at the different lesion sites), it was used as an anchor balloon to advance the guiding extension catheter over its shaft. The balloon anchoring technique was started in the proximal vessel, and then, as the guiding extension catheter was advanced into the RCA, the anchoring balloon technique was done in the mid and distal vessels. This allowed for incremental advancement of the guide extension catheter deeper into the RCA using a stepwise approach from the proximal to the distal part; this strategy enabled the guiding extension catheter to deeply intubate the RCA, facilitating the deployment of three DES (at high pressure (14-18 atmospheres)) from the distal to the proximal part of the vessel. The stent types (two Orsiro DES (Biotronik AG, Bülach, Switzerland) and one Resolute Onyx DES (Medtronic, Inc., Minneapolis, MN)) were chosen due to the size availability in our cardiac laboratory (Figures 8-11). All dilated segments were covered with a DES. Final angiography of the RCA showed good procedural results with no residual stenosis, no dissection, no thrombosis, and a thrombolysis in myocardial infarction (TIMI) III flow (Figure 12). Since all DES were deployed at high pressure, the operator did not perform post-stent deployment dilatation. Intravascular imaging was not done because intravascular ultrasound and optical coherent tomography are not available in our institution.

The patient tolerated the procedure well, was observed under cardiac monitoring for 24 hours, and was discharged the following day. There were no ECG changes over 24 hours. A repeat echocardiogram done before discharge showed a preserved ejection fraction at 55% with no evidence of wall motion abnormalities. Cardiac biomarkers were not ordered since they are not done routinely post-PCI in our institution. He was advised to continue dual antiplatelet therapy with aspirin 100 mg and clopidogrel 75 mg daily for one year, after which clopidogrel would be discontinued, and aspirin would be continued indefinitely. The patient was advised to perform exercise-stress cardiac scintigraphy in one year for follow-up on the PCI outcome.

## Discussion

When conventional methods such as an extra-support guiding catheter or a double-wire technique fail to deliver a DES to a distal calcified coronary lesion [1,2], the operator should use a guiding extension catheter to support DES placement [3,5]. If the guiding extension catheter does not provide adequate support, then the stepwise approach of guiding extension catheter advancement enhanced by repeated proximal to distal balloon anchoring becomes a valuable technique. This method leverages the anchoring force of an inflated balloon positioned distally or at the lesion site, providing the necessary stability for the incremental advancement of the guiding extension catheter over the coronary guidewire and balloon shaft through the coronary artery [6,7]. Our operators and staff are familiar with the guiding extension catheters and the balloon anchoring technique that permits advancement of the guiding catheter, but it was the first time the guiding extension catheter was advanced that deeply into a coronary artery. Other techniques, such as buddy balloon, anchor wire, and snare-assisted delivery, may help with DES delivery, but we encourage our operators to use the technique they are familiar with; in that way, the procedure will have less complication rate, such as vessel dissection and/or perforation.

In our case, this technique proved critical in facilitating the successful deployment of three DES in a severely calcified and diffusely diseased RCA. The repeated balloon anchoring provided sufficient mechanical advantage to overcome the resistance posed by the calcified lesions, ensuring that the guiding extension catheter could be advanced deep enough to allow for effective DES placement. Importantly, all sites where the balloon was inflated during the anchoring process were subsequently covered with DES to prevent restenosis, a crucial step to ensure long-term procedural success. Initially, the operator used a JR4 guiding catheter and did not shift to an extra-support guiding catheter to save time, as the patient started to experience chest pain. At this point, changing to an extra-backup guiding catheter will mean losing the Asahi Sion coronary wire already inside the RCA. Instead, a guiding extension catheter (Telescope Medtronic 6 French) was used, and with the anchoring technique, the operator was able to deploy the stents. This technique proved to be an excellent bailout strategy in this case, especially considering that the initial guiding catheter was not an extra-backup catheter and that the body wire technique had failed. Usually, using a buddy wire in calcified arteries will help stent deployment [1,2]. The application of this technique as a bailout option underscores its utility in complex PCI cases. By employing readily available equipment in the catheterization laboratory (a guiding extension catheter and a balloon), this method can be quickly and effectively executed, minimizing procedural time and patient discomfort. Moreover, its simplicity makes it an easy technique to learn and master, broadening its potential for widespread adoption. Also, mastering this technique is important to reduce complications. Of course, using intravascular imaging can help plan the PCI procedure, but our cardiac laboratory is not equipped with intracoronary imaging such as optical coherent tomography (OCT) or intravascular ultrasound, which would have been difficult to advance in this heavily calcified RCA. A lithotripsy or cutting balloons were not readily available; however, these bulky devices are also challenging to advance in this heavily calcified RCA. We are also not equipped with atherectomy devices (rotational atherectomy or laser). This limitation, which may apply to many laboratories globally, highlights the practicality of the balloon anchoring approach [2].

A pre-procedural CT coronary angiogram might have informed a more strategic initial approach, such as using an extra-support guiding catheter or planning for atherectomy. An atherectomy device, intravascular lithotripsy device, or cutting balloon could be prepared in our laboratory before the procedure when a pre-procedural CT scan shows heavy calcifications [1,2]. Because of the heavy calcification, all DES were deployed at high pressure (14-18 atmospheres) to ensure good DES opposition with the vessel wall. This, of course, like the balloon anchoring technique, carries a risk of vessel dissection and even perforation. In our cardiac laboratory, we dispose of multiple-sized stent grafts to treat these complications when they occur. The PCI results were optimal with no residual stenosis, no dissection, no thrombosis, and a TIMI III flow. By the end of the PCI, the patient was stable and had no chest pain. He was observed for 24 hours in the hospital under continuous ECG monitoring, and an echocardiography was done before discharge to ascertain that no left ventricular dysfunction had occurred. A follow-up at one year with exercise cardiac scintigraphy was planned.

## Conclusions

The stepwise balloon anchoring technique used to advance the guiding extension catheter into the coronary artery is a practical, effective, and reproducible method for delivering DES in complex, calcified coronary lesions when traditional methods fail. This method, utilizing only standard equipment available in most catheterization laboratories, offers a reliable solution when conventional techniques fail. The operator and the staff of the cardiac catheterization laboratory should be familiar with this procedure in order to minimize complications such as vessel dissection or perforation. Different-sized stent grafts should be available in the cardiac laboratory in case a dissection or perforation of the vessel occurs, and the operator should be well-trained in using these stent grafts to treat the vessel with these complications. Key considerations include avoiding balloon inflation in healthy coronary segments and ensuring that any segment where the balloon was inflated is covered with a DES to prevent restenosis. This case report highlights the technique's potential to enhance procedural success and patient outcomes in complex coronary interventions.
